# Long-term exposure to air pollution is associated with biological aging

**DOI:** 10.18632/oncotarget.12903

**Published:** 2016-10-25

**Authors:** Cavin K. Ward-Caviness, Jamaji C. Nwanaji-Enwerem, Kathrin Wolf, Simone Wahl, Elena Colicino, Letizia Trevisi, Itai Kloog, Allan C. Just, Pantel Vokonas, Josef Cyrys, Christian Gieger, Joel Schwartz, Andrea A. Baccarelli, Alexandra Schneider, Annette Peters

**Affiliations:** ^1^ Institute of Epidemiology II, Helmholtz Zentrum München, Neuherberg, Bavaria, Germany; ^2^ Department of Environmental Health, Harvard T.H. Chan School of Public Health, Boston, MA, USA; ^3^ Research Unit Molecular Epidemiology, Helmholtz Zentrum München, Neuherberg, Bavaria, Germany; ^4^ Department of Environmental Health Sciences, Mailman School of Public Health, Columbia University, New York, NY, USA; ^5^ Department of Global Health and Social Medicine, Harvard Medical School, Boston, MA, USA; ^6^ Department of Geography and Environmental Development, Ben-Gurion University of the Negev, Beer Sheva, Israel; ^7^ Department of Environmental Medicine and Public Health, Icahn School of Medicine at Mount Sinai, New York, NY, USA; ^8^ VA Normative Aging Study, Veterans Affairs Boston Healthcare System and the Department of Medicine, Boston University School of Medicine, Boston, MA, USA

**Keywords:** epigenetic aging, telomere length, biological aging, air pollution, black carbon, Gerotarget

## Abstract

Long-term exposure to air pollution is associated with age-related diseases. We explored the association between accelerated biological aging and air pollution, a potential mechanism linking air pollution and health. We estimated long-term exposure to PM_10_, PM_2.5_, PM_2.5_ absorbance/black carbon (BC), and NO_x_ via land-use regression models in individuals from the KORA F4 cohort. Accelerated biological aging was assessed using telomere length (TeloAA) and three epigenetic measures: DNA methylation age acceleration (DNAmAA), extrinsic epigenetic age acceleration (correlated with immune cell counts, EEAA), and intrinsic epigenetic age acceleration (independent of immune cell counts, IEAA). We also investigated sex-specific associations between air pollution and biological aging, given the published association between sex and aging measures. In KORA an interquartile range (0.97 μg/m^3^) increase in PM_2.5_ was associated with a 0.33 y increase in EEAA (CI = 0.01, 0.64; *P* = 0.04). BC and NO_x_ (indicators or traffic exposure) were associated with DNAmAA and IEAA in women, while TeloAA was inversely associated with BC in men. We replicated this inverse BC-TeloAA association in the Normative Aging Study, a male cohort based in the USA. A multiple phenotype analysis in KORA F4 combining all aging measures showed that BC and PM_10_ were broadly associated with biological aging in men. Thus, we conclude that long-term exposure to air pollution is associated with biological aging measures, potentially in a sex-specific manner. However, many of the associations were relatively weak and further replication of overall and sex-specific associations is warranted.

## INTRODUCTION

Long-term exposure to ambient air pollution is linked to a host of adverse age-related outcomes. Air pollution exposure is associated with cardiovascular disease [1–4], impaired cognitive function [5, 6], cancer [7–9], metabolic outcomes [10–12], and mortality [13–15]. All of the aforementioned air pollution associated outcomes are also associated with aging [16–18]. Multiple studies have explored the underlying biology of these associations and have linked air pollution exposure with DNA damage [19], epigenetic alterations [20–23], inflammation [24–26], and oxidative stress [25, 27, 28]. Given the overlap between air pollution and aging associated outcomes, accelerated biological aging may be another potential mechanism linking air pollution and adverse health outcomes.

Telomere length is one of the most widely used and validated measures of biological aging [29, 30], and long-term air pollution exposure has been associated with shortened telomeres, indicating an accelerated aging process [31–33], however a recent study has failed to replicate these associations [34]. Recently, summary measures representing epigenetic states have emerged as an accurate assessment of age and biological aging. Using methylation measured at select genetic loci, researchers have built “epigenetic clocks” that assess an individual's age and the deviation from their epigenetic age (DNAmAge) and chronological age [35, 36]. In particular the epigenetic age measure created by Horvath *et al* has been shown to be accurate across a wide range of tissues [35] and is associated with mortality [37] and metabolic outcomes [38].

A 2015 twin study compared the familial correlation of epigenetic age acceleration (DNAmAA) in monozygotic and dizygotic twins and concluded there was evidence for non-genetic, e.g. environmental, factors that influence DNAmAA [39]. However this study did not directly examine any environmental exposures. A 2016 study examined associations between epigenetic aging and air pollution exposure amongst men and determined there was a strong association between accelerated aging and both particulate matter < 2.5 μm in diameter (PM_2.5_) and black carbon (BC) [34]. Here we use the 4^th^ follow-up of the Cooperation for Health Research in the Region of Augsburg (KORA F4) cohort to associate accelerated biological aging with long-term air pollution exposure. We also compare these associations to those observed in the Normative Aging Study (NAS), an all-male cohort of veterans residing the Boston, Massachusetts metropolitan area, USA.

## RESULTS

**Figure 1:**
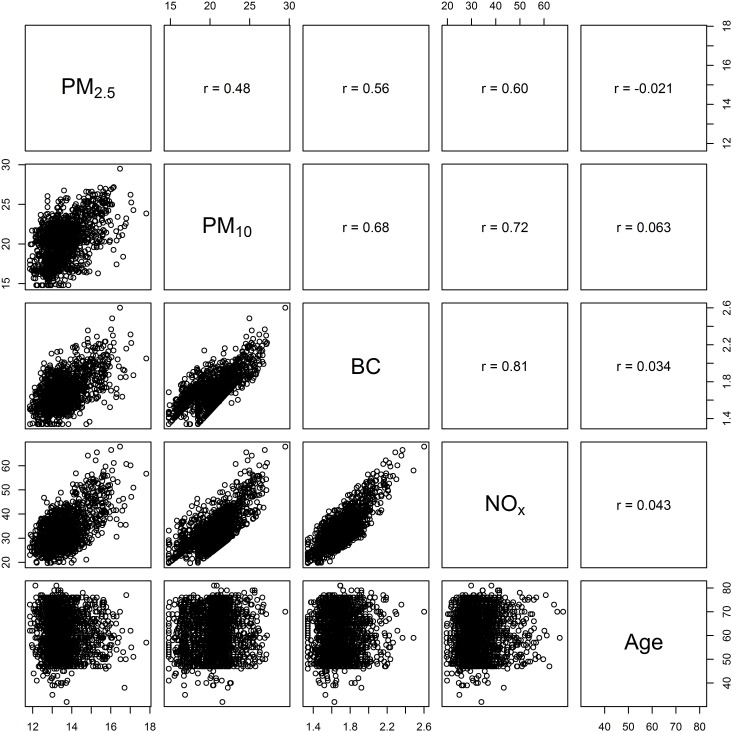
Pearson correlation between PM_2.5_, PM_10_, black carbon (BC), NO_2_, and NO_x_ in KORA Age is also shown to display the low correlation between the exposures and chronological age.

**Figure 2:**
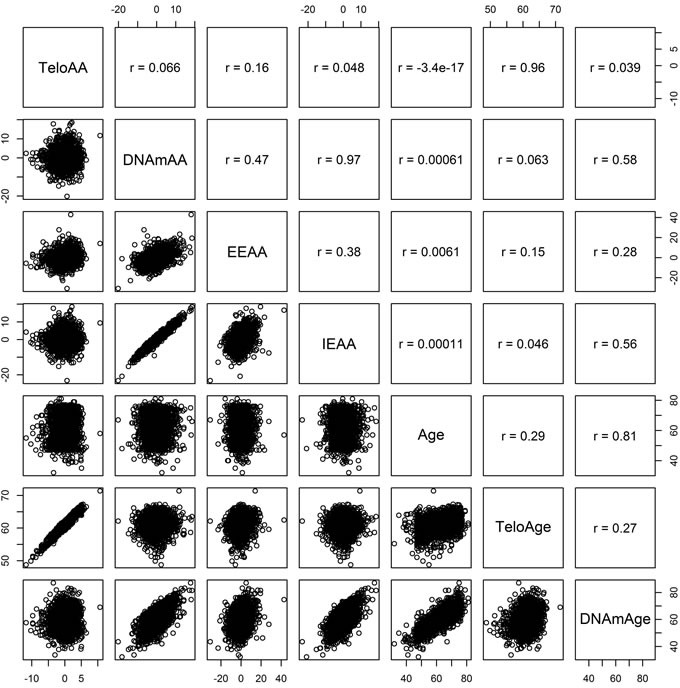
KORA Pearson correlations between telomere length based age acceleration (TeloAA), epigenetic age acceleration (DNAmAA), extrinsic epigenetic age acceleration (EEAA), intrinsic age acceleration (IEAA), chronological age (Age), telomere length estimated chronological age (TeloAge), and epigenetic age (DNAmAge)

Clinical characteristics of the KORA and NAS cohorts are given in Table 1. All biological age acceleration measures and air pollution exposures were independent of chronological age (Figures 1 and 2). We also note that telomere length is only weakly correlated with epigenetic aging measures indicating possible independence between these molecular aging assessments (Figure 2). The inter-quartile range (IQR) for the air pollution measures in KORA are as follows: PM_2.5_ (0.97 μg/m^3^), particulate matter < 10 μm in diameter (3.05 μg/m^3^; PM_10_), BC (0.20 μg/m^3^), mono-nitrogen oxides (8.39 μg/m^3^; NO_x_). IQRs for KORA are calculated from land-use regression models that represent the annual average pollution in the Augsburg, Germany region. For NAS the IQR for PM_2.5_ was 1.32 μg/m^3^ and for BC was 0.21 μg/m^3^. For NAS the PM_2.5_ and BC measurements represent the average over the year prior to blood draw. We use the terms “inverse associations” and “inversely associated” to indicate associations with a negative coefficient for the air pollution exposure being considered.

**Table 1:** Descriptive statistics for clinical covariates, air pollution exposures, and biological aging measures for KORA and NAS

	KORA (*N* = 1,777)		NAS (*N* = 496, Nobs = 734)	
	Mean	SD	Mean	SD
Age (y)	61	8.9	74	6.8
BMI (kg/m^2^)	28	4.8	27.9	4
LDL (mg/dL)	140	35		
Total cholesterol (mg/dL)			187.1	39.1
HDL (mg/dL)	57	15	48.4	13.1
Systolic BP (mm Hg)	120	19	127.7	17.4
Diastolic BP (mm Hg)	76	9.9	72.7	10.4
Pack-years	13	21	20.4	24.3
Physical Activity (METs)			13.6	22.2
	***N***	**Percent**	***N***	**Percent**
Sex (female)	855	48.4%	0	0%
Physical Activity (active)	1018	56.3%		
Smoking (ex-smoker)	772	43.4%	469	64%
Smoking (never)	747	42.0%	238	32%
Smoking (current)	256	14.4%	27	4.0%
Hypertension (yes)	350	19.7%	546	74%
**Air Pollution Exposures**	**Mean**	**SD**	**Mean**	**SD**
PM_2.5_ (μg/m^3^)	14	0.84	11.1	1
PM_10_ (μg/m^3^)	20	2.4		
BS (μg/m^3^)	1.7	0.17	0.53	0.2
NO_x_ (μg/m^3^)	33	7.1		
**Biological Aging Measures**	**Mean**	**SD**	**Mean**	**SD**
Telomere Length	1.8	0.31	1.3	0.7
DNAmAge (y)	59	7.7	74.5	8.2
TeloAge (y)	61	2.5	74.1	4.2
TeloAA (y)	1.9×10^−17^	2.4	1.5×10^−12^	1.5
DNAmAA (y)	−0.016	4.5	1	6.2
IEAA (y)	−0.027	4.3	0.3	5.3
EEAA (y)	−0.027	6	0.1	6.2

Pack-years was calculated as packs/day * years spent smoking as described in the methods. In NAS physical activity was measured as a continuous variable based on standardized questionnaires. BMI = body mass index; BC = black carbon; DNAmAA = epigenetic age acceleration; DNAmAge = epigenetic age; EEAA = extrinsic epigenetic age acceleration; HDL = high-density lipoprotein cholesterol; IEAA = intrinsic epigenetic age acceleration; LDL = low-density lipoprotein cholesterol; METs = metabolic equivalents; TeloAA = telomere length based age acceleration; TeloAge = age estimated via telomere length

### Combined sex associations

**Figure 3:**
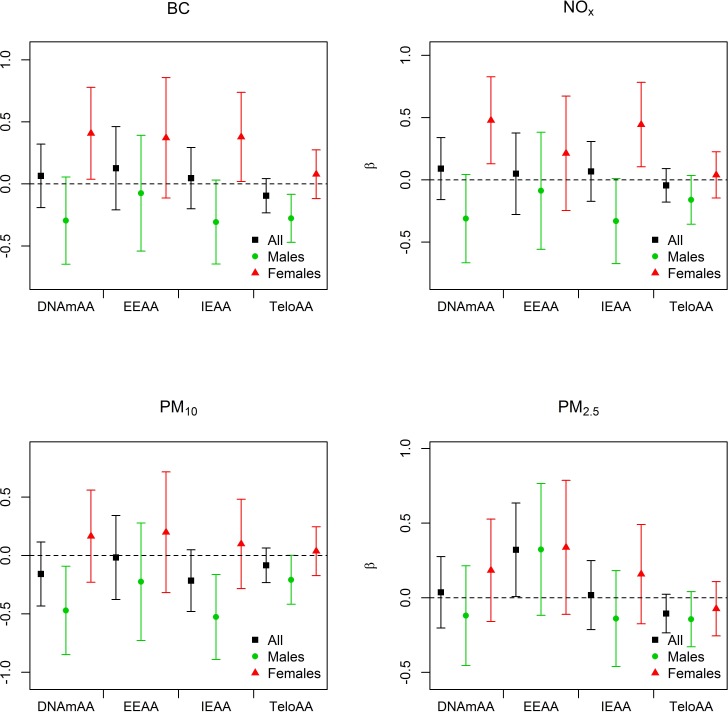
Associations between environmental exposures and measures of biological aging for full model in KORA Black is used for the combined-sex analyses, green associations when stratified on males, and red for the associations when stratified on females. The regression estimate (β) for each model is given on the y-axis and scaled to the inter-quartile range for each air pollution exposure. BC = black carbon, DNAmAA = epigenetic age acceleration, EEAA = extrinsic epigenetic age acceleration, IEAA = intrinsic epigenetic age acceleration, TeloAA = telomere length based age acceleration.

PM_2.5_ exposure was significantly associated with EEAA in KORA in all models (Table 2). The regression coefficient was stable across the models (β = 0.35 (basic); 0.35 (full)) indicating an independence of the association from the behavioral and clinical factors. For all of the models the regression coefficient was positive indicating that increased exposure to PM_2.5_ is associated with increased age acceleration. Neither IEAA, DNAmAA, nor TeloAA were associated with air pollution exposure in any of the combined sex models at the *P* < 0.05 level (Table 2, Figure 3, Supplemental Table 1). As expected given their strong correlation, results for NO_2_ were highly similar to that of NO_x_. However, NO_x_ associations were often larger in magnitude with smaller confidence intervals (Supplemental Table 2), reinforcing our reasoning to focus analyses on NO_x_. When adjusting for all air pollution exposures in a co-pollutant model PM_2.5_ was still associated with EEAA (β = 0.45; CI = 0.06, 0.85, *P* = 0.02).

**Table 2:** Results from the combined sex model for PM_2.5_

Biological Aging Measure	Exposure	Basic	Clinical	Behavioral	Full
TeloAA	PM_2.5_	β = −0.07 (CI = −0.20, 0.06; *P* = 0.29)	β = −0.09 (CI = −0.22, 0.04; *P* = 0.18)	β = −0.09 (CI = −0.22, 0.04; *P* = 0.18)	β = −0.11 (CI = −0.24, 0.02; *P* = 0.11)
DNAmAA	PM_2.5_	β =0.04 (CI = −0.20, 0.28; *P* = 0.73)	β =0.03 (CI = −0.21, 0.27; *P* = 0.81)	β =0.05 (CI = −0.19, 0.28; *P* = 0.71)	β = 0.04 (CI = −0.20, 0.28; *P* = 0.77)
EEAA	PM_2.5_	β =0.35 (CI = 0.04, 0.66; *P* = 0.03)*	β = 0.33 (CI = 0.024, 0.64; *P* = 0.04)*	β = 0.34 (CI = 0.02, 0.65; *P* = 0.04)*	β = 0.32 (CI = 0.007, 0.64; *P* = 0.045)*
IEAA	PM_2.5_	β =0.02 (CI = −0.21, 0.25; *P* = 0.89)	β = 0.007 (CI = −0.22, 0.24; *P* = 0.96)	β = 0.02 (CI = −0.21, 0.25; *P* = 0.84)	β = 0.02 (CI = −0.21, 0.25; *P* = 0.88)

Results for all exposures and all biological aging measures can be found in Supplemental Table 2.

* = *P* < 0.05 are marked. CI = 95% confidence interval. β = regression estimate scaled to the inter-quartile range for PM2.5 (0.97 μg/m^3^). DNAmAA = epigenetic age acceleration, EEAA = extrinsic epigenetic age acceleration, IEAA = intrinsic epigenetic age acceleration, TeloAA = Telomere length based age acceleration.

### Sex-specific associations

One possible reason for the lack of robust associations for IEAA, TeloAA, and DNAmAA is sex-specific associations that are attenuated in the combined-sex models. All of the age acceleration measures showed significant associations with sex, with the estimates indicating that males had a greater age acceleration (positive β values) than females with EEAA having the strongest association with sex (*P* = 1.3×10^−32^) (Supplemental Table 3). Chronological age was not associated with sex. To identify sex-specific associations we stratified KORA F4 by sex. We then tested for interactions to determine if there was a significant difference between the sex-specific associations.

There were three male-specific associations in the full model. PM_10_ was inversely associated with DNAmAA and IEAA in men, with IEAA having the strongest association (β = −0.53; CI = −0.89, −0.16; Table 3), and BC was inversely associated with TeloAA (β = −0.28; CI = −0.47, −0.08). We observed four female-specific associations, two with DNAmAA and two with IEAA for the BC and NO_x_ exposures. DNAmAA was more strongly associated with BC (β = 0.41; CI = 0.04, 0.78) and NO_x_ (β = 0.48; CI = 0.13, 0.83) than IEAA (Table 3). No exposure was significantly associated with aging amongst both men and women, however we observed that exposures were positively associated with biological aging measures amongst women and inversely associated amongst men. As before the sex-specific associations were largely independent of the clinical factor adjustment applied (Supplemental Table 4). Of the seven sex-specific associations, three remained significant in the co-pollutant models. Two of these were male-specific associations (PM_10_-IEAA and BC-TeloAA) and one was female-specific (NO_x_-IEAA) (Table 4). Of those three sex-specific associations that remained significant in the co-pollutant models, all had their effect estimate increase by 15.7% to 44.5%.

**Table 3:** Sex-specific results for full model

Biological Aging Measure	Exposure	Male	Female	Interaction
DNAmAA	PM_10_	β = −0.47 (CI = −0.85, −0.02; *P* = 0.02)*	β = 0.17 (CI = −0.23, 0.56; *P* = 0.41)	0.02
IEAA	PM_10_	β = −0.53 (CI = −0.89, −0.16; *P* = 0.005)**	β = 0.10 (CI = −0.28, 0.48; *P* = 0.61)	0.02
**TeloAA**	**BC**	β = −0.28 (CI = −0.47, −0.08;*P* = 0.005)**	**β = 0.08 (CI = −0.12, 0.27;***P*** = 0.44)**	**0.008**
DNAmAA	BC	β = −0.30 (CI = −0.65, 0.06; ***P***= 0.10)	β = 0.41 (CI = 0.037, 0.78; *P* = 0.03)*	0.01
**IEAA**	**BC**	**β = −0.31 (CI = −0.65, 0.03; *P* = 0.075)**	β = 0.38 (CI = 0.02, 0.74; *P* = 0.04)*	**0.0097**
**DNAmAA**	**NO_x_**	**β = −0.31 (CI = −0.67, 0.04; *P* = 0.09)**	β = 0.48 (CI = 0.13, 0.83; *P* = 0.008)**	**0.003**
**IEAA**	**NO_x_**	**β = −0.33 (CI = −0.67, 0.010; *P* = 0.057)**	β = 0.44 (CI = 0.11, 0.78; *P* = 0.01)*	**0.003**

Results for the male and female-specific full model analyses. Only those results with a *P* < 0.05 in one of the sexes are shown. Supplemental Table 3 has the complete results for all models and exposures. β = air pollution effect estimate scaled to the inter-quartile range for each exposure,

* = sex-specific *P* < 0.05,

** = sex-specific *P* < 0.01. Bolded associations are those with interaction *P* < 0.01. BC = black carbon, CI = 95% confidence interval, DNAmAA = epigenetic age acceleration, IEAA = intrinsic epigenetic age acceleration, TeloAA = Telomere length based age acceleration.

**Table 4:** Comparison of single- vs co-pollutant models for sex-specific associations

Sex	Exposure	Aging	Single β	Single CI	Single P	Co β	Co CI	Co P	β Diff	% Diff
Male	PM_10_	DNAmAA	−0.47	−0.85, −0.09	0.02	−0.51	−1.10, 0.04	0.07	−0.04	9.2%
**Male**	**PM_10_**	**IEAA**	**−0.53**	**−0.89, −0.16**	**0.005**	**−0.61**	**−1.10, −0.08**	**0.02**	**−0.08**	**15.7%**
**Male**	**BC**	**TeloAA**	**−0.28**	**−0.47, −0.08**	**0.005**	**−0.38**	**−0.72, −0.04**	**0.03**	**−0.10**	**37.0%**
Female	BC	DNAmAA	0.41	0.037, 0.78	0.03	0.15	−0.52, 0.81	0.66	−0.26	−63.6%
Female	BC	IEAA	0.38	0.02, 0.74	0.04	0.16	−0.48, 0.81	0.62	−0.21	−56.7%
Female	NO_x_	DNAmAA	0.48	0.13, 0.83	0.008	0.62	−0.03, 1.3	0.06	0.14	29.8%
**Female**	**NO_x_**	**IEAA**	**0.44**	**0.11, 0.78**	**0.01**	**0.64**	**0.02, 1.3**	**0.045**	**0.20**	**44.5%**

Columns labeled “Single” refer to the single pollutant models (model with only one air pollution exposure estimate) while columns labeled “Co” refer to the estimates from the co-pollutant models which contained all air pollution exposure estimates. The covariate adjustment followed the full model. Associations that were significant (*P* < 0.05) in the co-pollutant model are given in bold. β = effect estimate, CI = 95% confidence interval, *P* = *P*-value, β Diff = difference in effect estimates taken as the co-pollutant model β – single pollutant model β, % Diff = Percent difference in model effect estimates relative to the single pollutant model effect estimate.

### Multiple phenotype associations

We used a multiple phenotype association approach similar to some pleiotropy analyses undertaken in genetic epidemiology [40] to observe if any of the air pollution measures were associated with multiple biological aging measures. Our clinical covariate adjustment matched the full model used before. Given our observation of multiple sex-specific associations we did this for both the combined sex and sex-specific cohorts. When considering all four biological aging measures we observed evidence for associations with multiple phenotypes in the male stratified cohort, with PM_10_ (*P* = 0.005) and BC (*P* = 0.02) showing evidence for a broad association with the aging measures. We observed similar results when we excluded telomere length and restricted to just the three epigenetic aging measures, with males again showing the only association (PM_10_, *P* = 0.01).

### Associations in NAS

The NAS has previously published associations between epigenetic aging and air pollution amongst males [34]. Here we used a broader and slightly different array of aging measures. We retained their use of repeated measures as opposed to restricting to a single time point as this was the most powerful method in previous analyses. In this all male cohort, IEAA and TeloAA were both inversely associated with an IQR increase in PM_2.5_ in all models (Table 5). These inverse associations match what we observed in KORA F4 and each was greater in magnitude than the associations in the KORA F4 cohort. We did not observe an association between PM_2.5_ and either DNAmAA or EEAA in NAS. An IQR increase in BC was significantly, inversely associated with TeloAA in all models (Table 5), replicating the associations between TeloAA and BC amongst males in KORA F4. All NAS associations for PM_2.5_ and BC are given in Supplemental Table 5.

**Table 5:** Significant (*P* < 0.05) associations from NAS

Model	Exposure	Aging	Estimate	CI	*P*
Basic	PM2.5	IEAA	−0.37	−0.74, 0.00	0.049
Behavior	PM2.5	IEAA	−0.39	−0.76, −0.02	0.04
Clinical	PM2.5	IEAA	−0.40	−0.78, −0.02	0.04
Full	PM2.5	IEAA	−0.42	−0.80, −0.04	0.03
Basic	PM2.5	TeloAA	−0.54	−0.67, −0.41	<0.0001
Behavior	PM2.5	TeloAA	−0.54	−0.67, −0.41	<0.0001
Clinical	PM2.5	TeloAA	−0.49	−0.62, −0.36	<0.0001
Full	PM2.5	TeloAA	−0.49	−0.62, −0.36	<0.0001

Model	Exposure	Aging	Estimate		*P*
Basic	BC	TeloAA	−0.43	−0.55, −0.30	<0.0001
Behavior	BC	TeloAA	**−0.43**	**−0.56, −0.31**	<0.0001
Clinical	BC	TeloAA	**−0.40**	**−0.52, −0.28**	<0.0001
Full	BC	TeloAA	**−0.40**	**−0.53, −0.28**	<0.0001

Models follow the same naming convention as used for KORA. In NAS a continuous physical activity measure was used and total cholesterol was adjusted for while LDL was unavailable. All four aging measures examined in KORA were examined in NAS however only IEAA and TeloAA had significant associations. Supplemental Table 5 contains all NAS associations; BC = black carbon; IEAA = intrinsic epigenetic age acceleration; TeloAA = telomere-length based age acceleration

## DISCUSSION

We observed that exposure to ambient particulate matter is associated with epigenetic biomarkers of aging. A 0.97 μg/m^3^ increase in ambient PM_2.5_ was associated with a 0.32 - 0.35 y increase in EEAA indicating accelerated epigenetic aging. This association remained significant in our co-pollutant models indicating that it is independent of the other long-term air pollution exposures. We also observed multiple sex-specific associations with associations consistent with accelerated epigenetic aging amongst females, while we generally observed associations consistent with decelerated epigenetic aging for males (Figure 3, Table 3, Supplemental Table 4). The opposite directions of these associations may be responsible for the relative lack of associations in the combined-sex analysis. Exposure to NO_x_ and BC was associated with accelerated epigenetic aging amongst females. In men the direction of association for these exposures was in the opposite direction though not significant. A similar observation was noted for PM_10_ where men showed significant inverse associations with DNAmAA and IEAA while women showed positive, but non-significant, associations. Additionally BC was inversely associated with TeloAA amongst men in KORA.

PM_2.5_ is a measure of all particles less than 2.5 μm in diameter and is dominated by combustion particles, including both ones from primary sources (e.g. diesel engines) as well as secondary particles formed in the atmosphere *via* chemical reactions (e.g. sulfates). BC is primarily from diesel engines, but can also be produced from other combustion sources. Traffic exhaust contains NO_x_, a mixture of nitrogen oxides, which oxidizes into NO_2_ over timescales of hours. Emissions from diesel engines are substantially higher than from gasoline fueled vehicles. Hence, NO_x_ and BC are better representations of primary traffic emissions while PM_2.5_ and NO_2_ also include secondary pollutants. The stronger association with NO_x_ than with NO_2_, and the association of both NO_x_ and BC with some of the epigenetic biomarkers of aging in females suggest differences between traffic and non-traffic pollutants in the impact on epigenetic aging measures, and this should be addressed in future studies.

We re-examined our associations in NAS, a cohort of male veterans from the USA. As would be expected based on observations in KORA F4 we saw multiple inverse associations in this cohort with PM_2.5_ exposure inversely associated with TeloAA and IEAA. This matches the direction of association for the PM_2.5_-IEAA associations in KORA F4. Additionally, PM_10_ was significantly inversely associated with IEAA in KORA F4 males. Though PM_10_ was not assessed, in NAS it is primarily (up to 80%) composed of the smaller PM_2.5_ size fraction in this cohort making it likely that associations are similar for these related measures. BC was inversely associated with TeloAA, replicating the associations we observed in KORA. For NAS we used the subsection of the cohort with the clinical measures necessary for analysis. When we examined a slightly larger subset missing data on physical activity we still observed the BC-TeloAA associations however the PM_2.5_-IEAA associations were in the opposite direction (data not shown).

Thus, while we did replicate both associations these results still need to be carefully considered and further analyses in cohorts with even greater ethnic and exposure diversity should be undertaken to more firmly establish associations between molecular biomarkers of aging and air pollution.

Both accelerated and decelerated biological aging have been linked with negative health outcomes with accelerated aging linked to mortality and metabolic dysfunction [37, 38], and decelerated biological aging associated with the development of psychosocial stress [41]. To insure that the sex-specific associations in KORA F4 were not due to estimation inaccuracies in the biological aging measures, all measures were re-estimated in sex-stratified cohorts and the analyses re-run and the results were unchanged (data not shown).

### Air pollution and aging

Telomeres play an important role in inflammation-related pathways/cells [42–44], and inflammatory cells have the unique ability to extend their telomeres possibly to preserve replicative senescence [44]. It is possible that this short-term telomere extension is the reason that short-term exposure to air pollution is associated with increased telomere length [45, 46]. In long-term exposure studies, increased air pollution exposure has been generally associated with shorter telomere length [31–33, 46]. Coke oven workers, who are highly exposed to polycyclic aromatic hydrocarbons, were found to have significantly shorter telomere length than controls, and increased years of work in coke ovens was also associated with shorter telomere length [31]. In a study comparing truck drivers and office workers in Beijing, China, increased air pollution on the examination day was associated with longer telomere length, while 14-day average PM_10_ exposure was associated with a 10% shorter telomere length [47]. A 2015 study of 211 twins indicated that decreased residential traffic exposure of mothers was associated with longer placental telomere lengths [32] indicating that long-term traffic exposure during pregnancy may be passed down and affect biological aging in utero. A 2010 study of 165 non-smoking males indicated that long-term exposure to BC (often an indicator for automobile traffic) was associated with shorter telomere length [33]. This was the inverse of the associations observed in KORA. The differences could be due to differences in exposure assessment or in the fact that we calibrated our telomere based aging measure to the age of the cohorts whereas most previous studies simply used telomere length as their outcome. Although previous associations between BC and telomere length was strongest in the elderly (age > 75) subset, the age of the population is unlikely to be the main driver of these differences as we also observed inverse associations between BC and TeloAA in the NAS where the average age was 74 (SD = 6.8 y). With respect to epigenetic biomarkers of aging, there has been one previous study using the NAS cohort which showed positive associations between epigenetic age and 1-year PM_2.5_ and BC exposure [34].

Our study is one of the first to examine the effects of environmental exposures on two separate measures of biological aging, telomere length and epigenetic age. In our study and a previous that analyzed epigenetic and telomere length-based biomarkers of aging, no relationship was found between epigenetic aging and aging according to telomere length indicating that these processes might be independent [34]. In addition, our study extends the previous work by incorporating multiple air pollution and epigenetic aging measures and examining interactions by sex. All of the epigenetic age acceleration measures were strongly associated with sex (Supplemental Table 3) and we observed multiple female and male specific associations for these epigenetic age acceleration measures. For the epigenetic biomarkers of aging, the directions of associations were consistent across the exposures once the sex-stratifications are taken into account. For the male-specific KORA F4 associations we generally observed similar directions of effect in the all-male NAS cohort, with the BC-TeloAA association replication. However, these associations should still be carefully considered as substantial statistical evidence and replications are necessary to firmly validate sex-specific associations such as these. Further replications of the male-specific associations and replication of the female-specific associations should be performed for the sex-specific associations observed here..

In a multi-phenotype analysis in KORA F4, PM_10_ and BC were jointly associated with all biological aging measures in men. This indicates that these measures may be broadly associated with measures of biological aging while NO_x_ and PM_2.5_ may be more specific in their associations with biological aging measures.

### Biological aging and clinical outcomes

Telomeres are known to shorten over time, and accelerated shortening of telomeres has been associated with mortality [48–50]. More recently epigenetic age acceleration has also been associated with mortality [37]. Biological age acceleration measured by either telomere length or epigenetics has also been associated with a variety of other clinical outcomes including metabolic outcomes [38, 51], cancer [52], and atherosclerosis [51]. Although leukocyte telomere length is associated with a wide range of outcomes [51, 52], epigenetic aging has, for now, appeared to be more cell-type specific with DNAmAge associated with body mass index in liver cells but not in blood [38]. The strong associations between blood-based epigenetic biomarkers of age and mortality may be due to a causal association or result from systemic aging effects being reflected in the blood.

EEAA and IEAA are both associated with Parkinson's disease [53]. Semi-supercentenarians, individuals at least 105 years old, have a decreased IEAA as compared to controls (individuals age 52 - 75 with no centenarian parent) [54], and IEAA is associated with lung cancer incidence, particularly in older age groups, even when adjusting for smoking status [55]. Early-age telomere length in zebra fish has been associated with lifespan [56] strengthening the case for a causal role but further studies are needed to more fully explore underlying biological mechanisms.

### Strengths and limitations

This is one of the first studies to examine the association between environmental exposures and biological aging in a large population-based cohort. A previous study of epigenetic biomarkers of aging and air pollution using the NAS cohort observed positive associations between the epigenetic biomarkers and both PM_2.5_ and BC [34]. Our analysis included additional epigenetic aging biomarkers (e.g. IEAA and EEAA) than utilized in their study. In our replication, we used a slightly different subset of the NAS cohort and adjusted for confounders not assessed in the previous study. These differences likely account for the lack of association with DNAmAA (which is similar to the previous DNAmAge measured used in [34]) and the inverse associations observed with IEAA. No association between leukocyte telomere length and PM_2.5_ or BC was reported in the previous NAS publication, however our TeloAA measure calibrates leukocyte telomere length to the age of the cohort and thus may better assess associations with air pollution. Other previous studies used between 92 [31] and 211 [32] participants with either a case-control [31, 47] or twin study [32] design. With samples from 1,777 individuals drawn from the general population, our KORA F4 cohort is larger than any previous study. We were able to use the size and gender parity of our study to examine sex-specific associations and interactions. While some previous studies incorporated both sexes, none examined potential sex-based differences in the associations between environmental exposures and biological aging. Another strength of this study is the use of multiple measures of biological aging. Most previous studies only examined telomere length while this study used both telomere length and multiple DNA methylation based aging measures to assess biological aging. Our study was also able to incorporate multiple estimates of long-term air pollution exposures including both particulate matter and volatile compounds. Previous publications focused on particulate matter [34], land-use based indicators of traffic exposure [32], biomarkers of exposure [31], or only used a single ambient exposure for longer-term studies [47]. Thus this study builds upon and extends these previous analyses.

A limitation of this study is the inability to determine the precise number of years spent at the address. Exposures were assessed at the primary residence however we do not have the exact time at residence for the KORA F4 participants. Despite this we do know that all of the KORA F4 participants were also residents of Augsburg, Germany during the baseline survey (KORA S4) which took place 5-9 years prior to their follow-up examination. Thus, we expect these exposures to represent multi-year long-term exposures. The land use regression models used to assess air pollution exposure were developed for the 2008-2009 time period while KORA F4 was sampled in 2006-2007. Despite this these models have been shown to reflect historical exposures and similar modeling approaches have been shown to have a high correlation with long-term average exposures [57]. Another limitation of this study is that personal exposures could not be assessed and that epigenetics could only be measured in blood. Blood measures may not be the best tissue to assess epigenetic changes associated with air pollution exposure as the most directly affected tissues would be the esophagus and lung. Additionally, land use regression modeling may not perfectly correlate with personal exposure to air pollution, however land use regression modeling is a well-established technique, and the models developed for the ESCAPE study and implemented here have been validated and associated with a number of clinical outcomes [58–62]. A final limitation of this study is that many of these associations have thus far only been observed in the KORA cohort. While the NAS previously published their associations between BC and PM_2.5_ in men [34], research in this area is relatively new and our female-specific associations remain to be replicated.

In conclusion, multiple air pollution exposures are associated with biological aging, many of them in a sex-specific manner. Telomere-based and epigenetic measures of biological aging are associated with long-term exposure to air pollution and have distinct patterns of sex-specific associations. Further research is needed to connect accelerated aging and environmental exposures with clinical outcomes and to determine if patterns of sex-specific associations between environmental exposures and accelerated aging measures match patterns of sex-specific associations between environmental exposures and clinical outcomes.

## MATERIALS AND METHODS

### KORA F4

Participants for this study were taken from the follow-up to the fourth baseline survey of the Cooperation for Health Research in the Region of Augsburg (KORA F4). The baseline survey (N = 4,261) took place from October, 1999 to September, 2001 and 3,080 individuals participated in the follow-up (F4) survey from October, 2006 to May, 2008. The KORA F4 survey consisted of a lifestyle and medical history questionnaire as well as the collection of blood samples for later clinical chemistry and genomic analyses. The collection and details of this cohort have been previously published [63]. Of 3,080 individuals who participated in KORA F4, 1,799 had methylation data available. The collection and subsequent analysis of the KORA F4 cohort was approved by the Bavarian Medical Association Ethics Committee.

### Epigenetic aging measures

DNAmAge was calculated in KORA F4 using the online calculator as provided by the lab of Dr. Steve Horvath [35]. The details of the quality control for the methylation data as well as the use of the Horvath DNAmAge calculator in KORA F4 have been previously published [64]. Briefly, whole blood methylation was measured on 1,799 KORA F4 participants using the Infinium HumanMethylation450 BeachChip Array (Illumina). Background correction was performed by color channel and chip using the negative control probes available on the array. Background subtracted but unnormalized methylation values were used to calculate DNAmAge *via* the online calculator (https://dnamage.genetics.ucla.edu/). Unnormalized values were used as the online calculator performs its own internal normalization. Based on quality control metrics output by the online calculator we additionally filtered out samples according to the following quality control metrics: gender mismatch (N = 22); tissue type mismatch, i.e. non-blood estimated type, (N = 0); low correlation (r < 0.80) with an internal standard population (N = 0). This left 1,777 samples for subsequent analyses.

The DNAmAge calculator provides several estimates of epigenetic age acceleration. We focused on three measures from it. The first was the residuals after regressing DNAmAge on chronological age (DNAmAA). DNAmAA provides an estimate of age acceleration which has been shown to be valid for a variety of tissues [35]. The other two assessments of epigenetic aging adjust for blood immune cell counts and are thus blood specific. The epigenetic aging measure used was extrinsic epigenetic age acceleration (EEAA). This is a measure of age acceleration that adjusts for age as well as cell counts as determined by the Houseman [65] and Horvath cell count estimation methods [66] including: CD8 T-cells, CD4 T-cells, B lymphocytes, monocytes, and plasma blastocysts. EEAA is constructed to still strongly correlate with changes in naïve CD8+ T cells and exhausted CD8+ T cells [53], and could be interpreted as a modified Hannum epigenetic age [36] measure that even more strongly correlates with some immune cell counts. Thus EEAA assesses biological aging as associated with the immune system, particularly naïve and exhausted CD8+ T cells. The final epigenetic age acceleration measure was intrinsic epigenetic age acceleration (IEAA). IEAA adjusts for age and a broader set of cell counts including: naïve CD8+ T cells, exhausted CD8+ T cells, plasma B cells, CD4+ T cells, natural killer cells, monocytes, and granulocytes [53]. This more complete set of cell counts adjustments makes IEAA independent of the extrinsic cellular environment with respect to cell counts and thus more driven by the “intrinsic” intracellular environment.

### Telomere length assessment

Telomere length was measured in KORA *via* quantitative PCR [67] and expressed as the standardized ratio of the telomere repeat copy number to a single gene copy. This ratio was standardized to genomic DNA from the K562 line which was included on each assay. Full details on the method are presented elsewhere [68, 69]. To calculate telomere length based age acceleration (TeloAA) first a regression model was built to predict age based on telomere length (TeloAge). Telomeres are known to shorten over the lifetime and this was seen in our models where the regression coefficient of the model was negative. TeloAA was then estimated as the residuals from regressing chronological age on TeloAge. A positive TeloAA indicates an accelerated aging process from the perspective of telomere length. We observed only a weak correlation between telomere length and our epigenetic aging measures (Figure 2).

### Air pollution exposures

A total of four air pollutants were used for this analysis: particulate matter < 2.5μm in diameter (PM_2.5_), particulate matter < 10μm in diameter (PM_10_), PM_2.5_ absorbance (black carbon, BC), and mono-nitrogen oxides (NO_x_). NO_x_ is composed of several nitrogen oxides the primary of which is nitrogen dioxide (NO_2_). NO_2_ has been associated with several health outcomes [13, 70, 71] and existing air quality standards are often set relative to NO_2_. We observed strong correlation between NO_x_ and NO_2_ (Supplemental Figure 1), and despite their similar effect estimate, the CI for NO_x_ associations was often smaller than that for NO_2_. Therefore, rather than include both exposures we focused our analyses on NO_x_. All of the air pollutants were estimated *via* land use regression models developed as part of the multi-city ESCAPE study (www.escapeproject.eu). Full details on the modeling procedure including model validation and the formulation of the land-use regression models for the Augsburg, Germany region can be found elsewhere [61, 62]. After merging all clinical factors, aging measures, and air pollution exposures a total of 1,777 participants were available for this study.

### Statistical methods

Linear regression models implemented in R version 3.1.0 [72] were used to associate air pollution exposure with accelerated aging in the KORA F4 cohort. Air pollution measures were scaled to the inter-quartile range prior to all association analyses. Four models were used to explore the air pollution-biological aging associations. The initial model was a basic model adjusting for chronological age, chronological age^2^ and gender (sex). We included a quadratic age term to account for possibly non-linear associations with chronological age. Our second model was termed our “behavioral” model as it adjusted for age, age^2^ and sex but also included factors related to behavior such as physical activity (categorical: high *vs* low) and smoking both categorical (never, former, infrequent, frequent) and continuous (pack-years). Pack-years was calculated as packs/day (with 20 cigarettes to a pack) multiplied by years spent smoking. Our “clinical” model was so named because it adjusted for age, age^2^ and sex as well as the clinical variables body mass index (BMI, kg/m^2^), systolic blood pressure, diastolic blood pressure, low-density lipoprotein cholesterol (LDL, mg/dL), high-density lipoprotein cholesterol (mg/dL), and a binary indicator of hypertension. Hypertension was defined as systolic blood pressure above 90 mm Hg or diastolic blood pressure above 120 mm Hg. Our final model was a full model which included all terms from both the behavioral and clinical models. We tested for the independence of any significant air pollution measures by including multiple exposures in a co-pollutant model based on the full model. In this modeling approach all air pollution exposures were included in the same model and we observed the differences in effect estimates and confidence intervals as compared to the single pollutant models. As we observed significant correlation amongst the air pollution exposures (Figure 1), we used the variance inflation factor (VIF), as estimated *via* the “car” package [73], to determine the degree of multicollinearity. We used a conservative VIF cutoff of 4 to identify exposures showing substantial multicollinearity. This cutoff protects against substantial multicollinearity even though higher VIFs may not be purely indicative of multicollinearity [74]. As none of our air pollution exposures exceeded this VIF cutoff, we retained all in our co-pollutant models. The unit for all of the biological aging measures is years (y). Thus all of the regression estimates (β) represent biological aging acceleration/deceleration in number of years per inter-quartile range (IQR) increase in air pollution exposure. Given the correlation amongst some of the biological aging measures (Figure 2), we used a nominal significance threshold of *P* < 0.05 for all analyses.

To determine if any air pollution exposures show evidence for a broad association with multiple biological aging measures we used an approach similar to what is done when testing for pleiotropy in genetic association analyses [40]. In this approach we used the long-term air pollution estimates as the dependent variable and included each of our four biological aging measures in the set of predictor variables. We used the full model as the basis for our covariate adjustment. The “complete” model included all terms from the full model plus the four biological aging measures, while the “nested” model was the complete model lacking any biological aging measure terms. We tested for significance using a likelihood ratio test as implemented in the “lmtest” R package [75].

Telomere length is known to differ by sex [76–78]. Additionally, there have been multiple reports of sex differences in air pollution exposure associations [10, 79]. For these reasons we examined potential sex differences in associations between biological aging and air pollution as a secondary analysis, focusing on results from the full model. To examine sex-specific associations we stratified KORA F4 into female (N = 855) and male (N = 922) specific cohorts and re-analyzed the associations between air pollution and our biological aging measures. For any air pollution-aging measure pairs that showed a significant (*P* < 0.05) association in sex-stratified models, we formally tested for difference in the association between males and females by including an interaction term between air pollution and sex in the full model. The sex interaction term was considered significant at the *P* < 0.01 level.

### Associations in VA normative aging study

We compared our associations to those observed in the VA Normative Aging Study (NAS). NAS is a longitudinal cohort consisting of male volunteers residing in the Boston metropolitan area, USA. We retained all NAS participants with continued participation after 2000 as that is when PM_2.5_ measurements began in the study area. After excluding participants with missing values there were 496 left for analysis. As NAS is a longitudinal study some individuals had multiple assessments available (N_obs_ = 734). We used all available measures as this was the most powerful method in the previous analysis using NAS[34].

Leukocyte telomere length was assessed via quantitative real time polymerase chain reaction. Methylation was assessed via the 450K chip and epigenetic aging measures assessed via the epigenetic aging online calculator as done in KORA. Descriptions of the NAS cohort telomere length and methylation assessment have been previously published [80].

All statistical analyses were done using R v3.1.1 [72]. A generalized linear mixed effects model was used with a random intercept for each participant to account for the multiple observations per individual [34]. A spatiotemporal land-use regression model was used to assess BC at each resident's address [81] while PM_2.5_ was assessed *via* a hybrid model that combined satellite aerial optical depth measurements with land-use regression. PM_2.5_ measurements were available on a 1×1 km grid [82, 83]. Both PM_2.5_ and BC were measured in μg/m^3^ and scaled to their respective IQRs of 1.32 μg/m^3^ and 0.21 μg/m^3^. Air pollution measurements in NAS represent the average air pollution in the year prior to the blood draw used for analysis. LDL was unavailable in NAS and thus total cholesterol was adjusted for in the models. Physical activity was assessed as the metabolic equivalents per week based on validated and standardized questionnaires [84] and was used as a continuous variable in the models. All clinical covariates for the NAS are available in Table 1. NAS was approved by the Harvard T.H. Chan School of Public Health and Veterans Affairs (VA) Institutional Review Board (IRB). Records of written and informed consent from each participant were provided to the VA IRB.

## SUPPLEMENTARY MATERIALS FIGURES AND TABLES


