# Supplementary material for: Long-term exposure to air pollution is associated with biological aging
Source: Oncotarget. 2016 Oct 25;7(46):74510–25. doi: 10.18632/oncotarget.12903 (PMC5342683; doi:10.18632/oncotarget.12903)
Supplement: Supplementary file 1 [file oncotarget-07-74510-s001.pdf]

Long-term exposure to air pollution is associated with biological aging

Supplemental Material

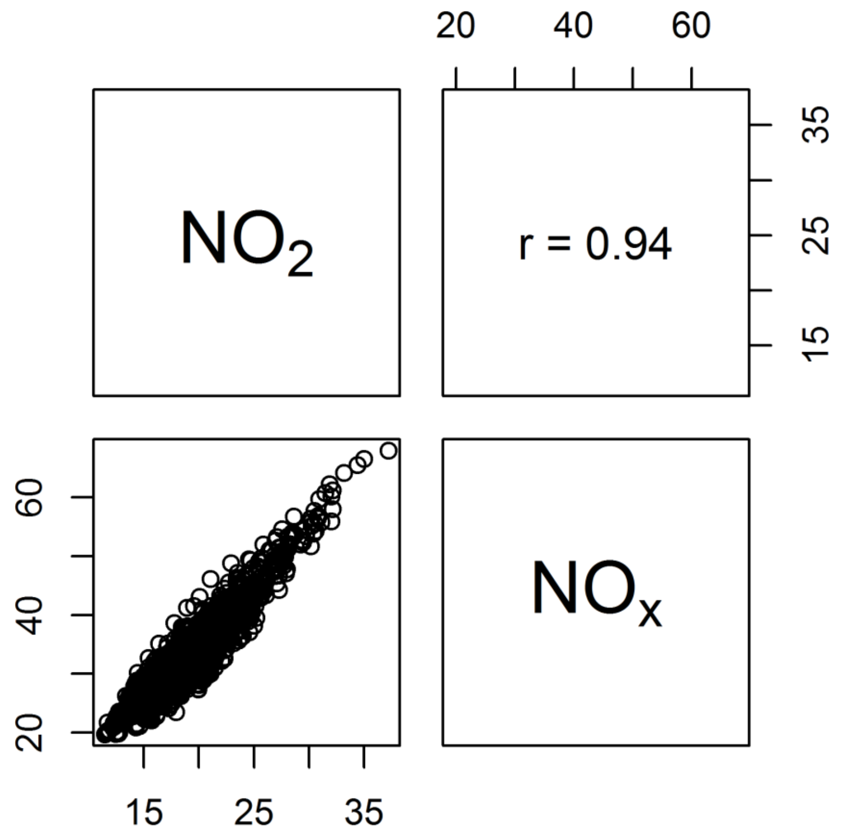

Supplemental Figure 1: Correlation between NO_2 and NO_x in KORA. Numbers on the sides represent the concentration of NO_2 and NO_x in µg/m^3

Supplemental Table 1: Results from the combined sex model. * = P < 0.05. ** = P < 0.01; BC = black carbon

Biological Age Acceleration Measure	Exposure	Basic	Clinical	Behavioral	Full
TeloAA	$PM_{2.5}$	0.09 (CI = -0.04, 0.22; P = 0.18)	0.10 (CI = -0.03, 0.24; P = 0.13)	0.11 (CI = -0.03, 0.24; P = 0.11)	0.12 (CI = -0.016, 0.25; P = 0.09)
DNAmAA	$PM_{2.5}$	0.04 (CI = -0.19, 0.28; P = 0.71)	0.031 (CI = -0.21, 0.27; P = 0.8)	0.047 (CI = -0.19, 0.29; P = 0.7)	0.038 (CI = -0.2, 0.28; P = 0.76)
EEAA	$PM_{2.5}$	0.36 (CI = 0.05, 0.67; P = 0.02)*	0.34 (CI = 0.031, 0.65; P = 0.03)*	0.34 (CI = 0.029, 0.66; P = 0.03)*	0.33 (CI = 0.012, 0.64; P = 0.04)*
IEAA	$PM_{2.5}$	0.02 (CI = -0.21, 0.25; P = 0.86)	0.009 (CI = -0.22, 0.24; P = 0.94)	0.027 (CI = -0.2, 0.26; P = 0.82)	0.02 (CI = -0.21, 0.25; P = 0.87)
TeloAA	PM_{10}	0.07 (CI = -0.084, 0.22; P = 0.39)	0.072 (CI = -0.079, 0.22; P = 0.35)	0.078 (CI = -0.075, 0.23; P = 0.32)	0.084 (CI = -0.07, 0.24; P = 0.29)
DNAmAA	PM_{10}	-0.15 (CI = -0.42, 0.12; P = 0.27)	-0.17 (CI = -0.44, 0.1; P = 0.23)	-0.15 (CI = -0.42, 0.12; P = 0.29)	-0.16 (CI = -0.43, 0.12; P = 0.26)
EEAA	PM_{10}	0.002 (CI = -0.35, 0.35; P = 0.99)	$7.4x10^{-5}$ (CI = -0.35, 0.35; P = 1)	-0.021 (CI = -0.38, 0.34; P = 0.91)	-0.02 (CI = -0.38, 0.34; P = 0.93)
IEAA	PM_{10}	-0.21 (CI = -0.47, 0.05; P = 0.12)	-0.22 (CI = -0.48, 0.04; P = 0.1)	-0.21 (CI = -0.47, 0.06; P = 0.12)	-0.22 (CI = -0.48, 0.05; P = 0.11)
TeloAA	BC	0.08 (CI = -0.06, 0.23; P = 0.24)	0.09 (CI = -0.06, 0.23; P = 0.24)	0.09 (CI = -0.05, 0.23; P = 0.21)	0.09 (CI = -0.05, 0.23; P = 0.21)
DNAmAA	BC	0.05 (CI = -0.21, 0.3; P = 0.73)	0.04 (CI = -0.21, 0.3; P = 0.74)	0.06 (CI = -0.19, 0.32; P = 0.64)	0.06 (CI = -0.19, 0.32; P = 0.62)
EEAA	BC	0.12 (CI = -0.21, 0.45; P = 0.48)	0.13 (CI = -0.2, 0.46; P = 0.45)	0.11 (CI = -0.23, 0.44; P = 0.53)	0.12 (CI = -0.21, 0.46; P = 0.47)
IEAA	BC	0.02 (CI = -0.22, 0.27; P = 0.86)	0.02 (CI = -0.22, 0.27; P = 0.85)	0.04 (CI = -0.21, 0.29; P = 0.75)	0.05 (CI = -0.2, 0.29; P = 0.72)
TeloAA	NO_x	0.04 (CI = -0.10, 0.17; P = 0.59)	0.04 (CI = -0.10, 0.18; P = 0.55)	0.03 (CI = -0.11, 0.17; P = 0.65)	0.03 (CI = -0.11, 0.17; P = 0.63)
DNAmAA	NO_x	0.09 (CI = -0.16, 0.33; P = 0.49)	0.08 (CI = -0.17, 0.32; P = 0.54)	0.09 (CI = -0.16, 0.34; P = 0.47)	0.09 (CI = -0.16, 0.34; P = 0.49)
EEAA	NO_x	0.08 (CI = -0.24, 0.4; P = 0.62)	0.07 (CI = -0.25, 0.39; P = 0.68)	0.05 (CI = -0.28, 0.38; P = 0.76)	0.05 (CI = -0.28, 0.37; P = 0.79)
IEAA	NO_x	0.06 (CI = -0.18, 0.3; P = 0.61)	0.06 (CI = -0.18, 0.29; P = 0.64)	0.06 (CI = -0.18, 0.3; P = 0.6)	0.07 (CI = -0.17, 0.31; P = 0.59)

Supplemental Table 2: Associations for NO_2 and NO_x for Full model. BC = black carbon; CI Width= width of the confidence interval; EEAA = extrinsic epigenetic aging; IEAA = intrinsic epigenetic aging; Stratification = sex-stratification used

Biological Aging Measure	Exposure	Full Model	CI Width	Stratification
DNAmAA	NO_2	0.042 (CI = -0.24, 0.32; P = 0.77)	0.56	Combined-Sex
DNAmAA	NO_x	0.09 (CI = -0.16, 0.34; P = 0.48)	0.50	Combined-Sex
EEAA	NO_2	0.072 (CI = -0.29, 0.44; P = 0.7)	0.73	Combined-Sex
EEAA	NO_x	0.049 (CI = -0.28, 0.38; P = 0.77)	0.66	Combined-Sex
IEAA	NO_2	0.014 (CI = -0.25, 0.28; P = 0.92)	0.53	Combined-Sex
IEAA	NO_x	0.067 (CI = -0.17, 0.31; P = 0.58)	0.48	Combined-Sex
TeloAA	NO_2	-0.041 (CI = -0.19, 0.11; P = 0.59)	0.3	Combined-Sex
TeloAA	NO_x	-0.045 (CI = -0.18, 0.09; P = 0.52)	0.27	Combined-Sex
DNAmAA	NO_2	-0.35 (CI = -0.74, 0.04; P = 0.079)	0.78	Males
DNAmAA	NO_x	-0.31 (CI = -0.67, 0.043; P = 0.085)	0.71	Males
EEAA	NO_2	-0.14 (CI = -0.65, 0.38; P = 0.61)	1.03	Males
EEAA	NO_x	-0.088 (CI = -0.56, 0.38; P = 0.71)	0.94	Males
IEAA	NO_2	-0.39 (CI = -0.77, -0.016; P = 0.041)	0.75	Males
IEAA	NO_x	-0.33 (CI = -0.67, 0.0095; P = 0.057)	0.68	Males
TeloAA	NO_2	-0.11 (CI = -0.32, 0.11; P = 0.33)	0.43	Males
TeloAA	NO_x	-0.16 (CI = -0.36, 0.035; P = 0.11)	0.40	Males
DNAmAA	NO_2	0.43 (CI = 0.04, 0.82; P = 0.031)	0.78	Females
DNAmAA	NO_x	0.48 (CI = 0.13, 0.83; P = 0.0075)	0.7	Females
EEAA	NO_2	0.29 (CI = -0.22, 0.8; P = 0.27)	1.02	Females
EEAA	NO_x	0.21 (CI = -0.25, 0.67; P = 0.36)	0.92	Females
IEAA	NO_2	0.4 (CI = 0.025, 0.78; P = 0.037)	0.76	Females
IEAA	NO_x	0.44 (CI = 0.11, 0.78; P = 0.01)	0.67	Females
TeloAA	NO_2	0.002 (CI = -0.21, 0.21; P = 0.99)	0.42	Females
TeloAA	NO_x	0.039 (CI = -0.15, 0.23; P = 0.68)	0.38	Females

Supplemental Table 3: Association between sex and accelerated aging measures and chronological age. Sex was significantly associated with epigenetic age acceleration measures, however was not significantly associated with chronological age or TeloAA. β = regression estimate of sex on "Aging Measure" with females as the "baseline" gender, CI = 95% Confidence Interval

Aging Measure	β	CI	P
TeloAA	0.9	0.67, 1.12	$4.70x10^{-15}$
DNAmAA	1.25	0.84, 1.66	$3.13x10^{-09}$
IEAA	1.09	0.69, 1.49	$9.50x10^{-08}$
EEAA	3.35	2.81, 3.89	$1.30x10^{-32}$
Age	0.64	-0.18, 1.47	0.13
DNAmAge	1.25	0.99, 2.41	$2.73x10^{-6}$
TeloAge	0.95	0.72, 1.19	$1.96x10^{-15}$

Supplemental Table 4: Results from the sex-specific models. BC = black carbon; DNAmAA = epigenetic age acceleration; EEAA = extrinsic epigenetic age acceleration; IEAA = intrinsic epigenetic age acceleration; TeloAA = telomere based age acceleration * = P < 0.05, ** = P < 0.01

Males					
Aging Measure	Exposure	Basic	Behavioral	Clinical	Full
TeloAA	$PM_{2.5}$	β = -0.10 (CI = -0.29, 0.078; P = 0.26)	β = -0.12 (CI = -0.31, 0.058; P = 0.18)	β = -0.13 (CI = -0.31, 0.056; P = 0.17)	β = -0.14 (CI = -0.33, 0.041; P = 0.13)
DNAmAA	$PM_{2.5}$	β = -0.15 (CI = -0.47, 0.18; P = 0.37)	β = -0.14 (CI = -0.47, 0.19; P = 0.41)	β = -0.14 (CI = -0.47, 0.19; P = 0.41)	β = -0.12 (CI = -0.45, 0.21; P = 0.48)
EEAA	$PM_{2.5}$	β = 0.31 (CI = -0.12, 0.74; P = 0.16)	β = 0.3 (CI = -0.13, 0.74; P = 0.17)	β = 0.33 (CI = -0.11, 0.76; P = 0.15)	β = 0.32 (CI = -0.12, 0.77; P = 0.15)
IEAA	$PM_{2.5}$	β = -0.17 (CI = -0.49, 0.14; P = 0.28)	β = -0.16 (CI = -0.48, 0.16; P = 0.32)	β = -0.16 (CI = -0.48, 0.16; P = 0.32)	β = -0.14 (CI = -0.46, 0.18; P = 0.4)
TeloAA	PM_{10}	β = -0.21 (CI = -0.41, -0.0037; P = 0.046)*	β = -0.19 (CI = -0.4, 0.013; P = 0.066)	β = -0.22 (CI = -0.43, -0.014; P = 0.037)*	β = -0.21 (CI = -0.42, 0.0016; P = 0.052)
DNAmAA	PM_{10}	β = -0.49 (CI = -0.86, -0.13; P = 0.0083)**	β = -0.48 (CI = -0.85, -0.11; P = 0.011)*	β = -0.49 (CI = -0.87, -0.12; P = 0.011)*	β = -0.47 (CI = -0.85, -0.092; P = 0.015)*
EEAA	PM_{10}	β = -0.21 (CI = -0.7, 0.28; P = 0.4)	β = -0.21 (CI = -0.7, 0.28; P = 0.41)	β = -0.23 (CI = -0.73, 0.27; P = 0.37)	β = -0.22 (CI = -0.73, 0.28; P = 0.38)
IEAA	PM_{10}	β = -0.56 (CI = -0.91, -0.21; P = 0.0019)**	β = -0.54 (CI = -0.9, -0.19; P = 0.0028)**	β = -0.55 (CI = -0.91, -0.19; P = 0.003)**	β = -0.53 (CI = -0.89, -0.16; P = 0.0047)**
TeloAA	BC	β = -0.27 (CI = -0.46, -0.08; P = 0.0055)**	β = -0.26 (CI = -0.46, -0.074; P = 0.0067)**	β = -0.29 (CI = -0.48, -0.094; P = 0.0037)**	β = -0.28 (CI = -0.47, -0.084; P = 0.005)**
DNAmAA	BC	β = -0.28 (CI = -0.63, 0.059; P = 0.1)	β = -0.29 (CI = -0.63, 0.054; P = 0.099)	β = -0.29 (CI = -0.64, 0.055; P = 0.099)	β = -0.3 (CI = -0.65, 0.056; P = 0.1)
EEAA	BC	β = -0.072 (CI = -0.53, 0.38; P = 0.76)	β = -0.079 (CI = -0.54, 0.38; P = 0.73)	β = -0.075 (CI = -0.54, 0.39; P = 0.75)	β = -0.075 (CI = -0.54, 0.39; P = 0.75)
IEAA*	BC	β = -0.31 (CI = -0.64, 0.024; P = 0.069)	β = -0.31 (CI = -0.64, 0.02; P = 0.066)	β = -0.31 (CI = -0.64, 0.031; P = 0.075)	β = -0.31 (CI = -0.65, 0.031; P = 0.075)
TeloAA	NO_x	β = -0.16 (CI = -0.35, 0.031; P = 0.1)	β = -0.16 (CI = -0.35, 0.03; P = 0.099)	β = -0.16 (CI = -0.36, 0.033; P = 0.1)	β = -0.16 (CI = -0.36, 0.035; P = 0.11)
DNAmAA	NO_x	β = -0.31 (CI = -0.66, 0.03; P = 0.074)	β = -0.31 (CI = -0.66, 0.038; P = 0.081)	β = -0.33 (CI = -0.68, 0.024; P = 0.068)	β = -0.31 (CI = -0.67, 0.043; P = 0.085)
EEAA	NO_x	β = -0.045 (CI = -0.5, 0.41; P = 0.85)	β = -0.069 (CI = -0.53, 0.39; P = 0.77)	β = -0.072 (CI = -0.54, 0.4; P = 0.76)	β = -0.088 (CI = -0.56, 0.38; P = 0.71)

IEAA	NO_x	β = -0.35 (CI = -0.68, -0.015; P = 0.041)*	β = -0.34 (CI = -0.67, -0.00092; P = 0.05)	β = -0.35 (CI = -0.69, -0.014; P = 0.042)*	β = -0.33 (CI = -0.67, 0.0095; P = 0.057)
Females					
Biological Aging Measure	Exposure	Basic	Behavioral	Clinical	Full
TeloAA	$PM_{2.5}$	β = -0.044 (CI = -0.22, 0.14; P = 0.63)	β = -0.057 (CI = -0.24, 0.12; P = 0.54)	β = -0.061 (CI = -0.24, 0.12; P = 0.51)	β = -0.074 (CI = -0.26, 0.11; P = 0.43)
DNAmAA	$PM_{2.5}$	β = 0.22 (CI = -0.12, 0.57; P = 0.2)	β = 0.2 (CI = -0.15, 0.54; P = 0.26)	β = 0.2 (CI = -0.14, 0.54; P = 0.25)	β = 0.18 (CI = -0.16, 0.53; P = 0.29)
EEAA	$PM_{2.5}$	β = 0.4 (CI = -0.043, 0.85; P = 0.077)	β = 0.39 (CI = -0.058, 0.83; P = 0.089)	β = 0.35 (CI = -0.1, 0.8; P = 0.13)	β = 0.34 (CI = -0.11, 0.79; P = 0.14)
IEAA	$PM_{2.5}$	β = 0.2 (CI = -0.14, 0.53; P = 0.24)	β = 0.17 (CI = -0.16, 0.51; P = 0.31)	β = 0.18 (CI = -0.15, 0.51; P = 0.29)	β = 0.16 (CI = -0.17, 0.49; P = 0.35)
TeloAA	PM_{10}	β = 0.076 (CI = -0.13, 0.28; P = 0.46)	β = 0.048 (CI = -0.16, 0.25; P = 0.64)	β = 0.064 (CI = -0.14, 0.27; P = 0.55)	β = 0.038 (CI = -0.17, 0.25; P = 0.73)
DNAmAA	PM_{10}	β = 0.18 (CI = -0.21, 0.57; P = 0.38)	β = 0.13 (CI = -0.26, 0.52; P = 0.5)	β = 0.2 (CI = -0.19, 0.6; P = 0.31)	β = 0.17 (CI = -0.23, 0.56; P = 0.41)
EEAA	PM_{10}	β = 0.21 (CI = -0.3, 0.72; P = 0.42)	β = 0.21 (CI = -0.29, 0.72; P = 0.41)	β = 0.18 (CI = -0.33, 0.7; P = 0.49)	β = 0.2 (CI = -0.32, 0.72; P = 0.45)
IEAA*	PM_{10}	β = 0.13 (CI = -0.25, 0.51; P = 0.5)	β = 0.092 (CI = -0.29, 0.47; P = 0.64)	β = 0.14 (CI = -0.25, 0.52; P = 0.49)	β = 0.1 (CI = -0.28, 0.48; P = 0.61)
TeloAA*	BC	β = 0.091 (CI = -0.1, 0.28; P = 0.36)	β = 0.08 (CI = -0.12, 0.27; P = 0.42)	β = 0.087 (CI = -0.11, 0.28; P = 0.38)	β = 0.078 (CI = -0.12, 0.27; P = 0.44)
DNAmAA	BC	β = 0.36 (CI = -0.0039, 0.73; P = 0.053)	β = 0.36 (CI = -0.0062, 0.73; P = 0.054)	β = 0.4 (CI = 0.032, 0.77; P = 0.034)*	β = 0.41 (CI = 0.037, 0.78; P = 0.031)*
EEAA	BC	β = 0.33 (CI = -0.15, 0.81; P = 0.18)	β = 0.37 (CI = -0.11, 0.85; P = 0.13)	β = 0.32 (CI = -0.16, 0.81; P = 0.19)	β = 0.37 (CI = -0.11, 0.86; P = 0.13)
IEAA*	BC	β = 0.34 (CI = -0.015, 0.7; P = 0.061)	β = 0.35 (CI = -0.013, 0.71; P = 0.059)	β = 0.37 (CI = 0.013, 0.73; P = 0.043)*	β = 0.38 (CI = 0.02, 0.74; P = 0.039)*
TeloAA	NO_x	β = 0.052 (CI = -0.13, 0.23; P = 0.57)	β = 0.036 (CI = -0.15, 0.22; P = 0.69)	β = 0.052 (CI = -0.13, 0.24; P = 0.58)	β = 0.039 (CI = -0.15, 0.23; P = 0.68)
DNAmAA*	NO_x	β = 0.44 (CI = 0.092, 0.78; P = 0.013)*	β = 0.43 (CI = 0.08, 0.77; P = 0.016)*	β = 0.48 (CI = 0.13, 0.83; P = 0.007)**	β = 0.48 (CI = 0.13, 0.83; P = 0.0075)**
EEAA	NO_x	β = 0.22 (CI = -0.23, 0.67; P = 0.35)	β = 0.23 (CI = -0.22, 0.68; P = 0.32)	β = 0.19 (CI = -0.27, 0.65; P = 0.42)	β = 0.21 (CI = -0.25, 0.67; P = 0.36)
IEAA*	NO_x	β = 0.42 (CI = 0.081, 0.75; P = 0.015)*	β = 0.41 (CI = 0.074, 0.75; P = 0.017)*	β = 0.45 (CI = 0.11, 0.78; P = 0.01)*	β = 0.44 (CI = 0.11, 0.78; P = 0.01)*

Supplemental Table 5: Associations between $PM_{2.5}$ and BC for the Normative Aging Study

Model	Exposure	Aging	Estimate	SE	T	P	Low 95 CI	High 95 CI
Basic	PM2.5	DNAmAA	-0.19	0.20	-0.93	0.35	-0.58	0.21
Behavior	PM2.5	DNAmAA	-0.22	0.20	-1.12	0.26	-0.62	0.17
Clinical	PM2.5	DNAmAA	-0.26	0.20	-1.26	0.21	-0.66	0.14
Full	PM2.5	DNAmAA	-0.30	0.21	-1.46	0.15	-0.70	0.10
Basic	PM2.5	EEAA	-0.27	0.19	-1.45	0.15	-0.63	0.10
Behavior	PM2.5	EEAA	-0.26	0.19	-1.41	0.16	-0.63	0.10
Clinical	PM2.5	EEAA	-0.27	0.19	-1.40	0.16	-0.64	0.11
Full	PM2.5	EEAA	-0.26	0.19	-1.35	0.18	-0.64	0.12
Basic	PM2.5	IEAA	-0.37	0.19	-1.98	0.05	-0.74	0.00
Behavior	PM2.5	IEAA	-0.39	0.19	-2.08	0.04	-0.76	-0.02
Clinical	PM2.5	IEAA	-0.40	0.19	-2.07	0.04	-0.78	-0.02
Full	PM2.5	IEAA	-0.42	0.19	-2.18	0.03	-0.80	-0.04
Basic	PM2.5	TeloAA	-0.54	0.07	-7.93	<0.0001	-0.67	-0.41
Behavior	PM2.5	TeloAA	-0.54	0.07	-7.91	<0.0001	-0.67	-0.41
Clinical	PM2.5	TeloAA	-0.49	0.07	-7.27	<0.0001	-0.62	-0.36
Full	PM2.5	TeloAA	-0.49	0.07	-7.23	<0.0001	-0.62	-0.36
**Model**	**Exposure**	**Aging**	**Estimate**	**SE**	**T**	**P**	**Low 95 CI**	**High 95 CI**
Basic	BC	DNAmAA	0.00	0.28	0.01	0.99	-0.54	0.55
Behavior	BC	DNAmAA	-0.04	0.28	-0.13	0.90	-0.58	0.51
Clinical	BC	DNAmAA	-0.07	0.28	-0.26	0.80	-0.63	0.49
Full	BC	DNAmAA	-0.11	0.28	-0.40	0.69	-0.67	0.45
Basic	BC	EEAA	0.32	0.27	1.20	0.23	-0.21	0.85
Behavior	BC	EEAA	0.31	0.27	1.14	0.25	-0.22	0.83
Clinical	BC	EEAA	0.32	0.28	1.18	0.24	-0.22	0.87
Full	BC	EEAA	0.30	0.28	1.08	0.28	-0.25	0.84
Basic	BC	IEAA	0.38	0.24	1.57	0.12	-0.10	0.86
Behavior	BC	IEAA	0.35	0.24	1.44	0.15	-0.13	0.82
Clinical	BC	IEAA	0.39	0.25	1.59	0.11	-0.09	0.88
Full	BC	IEAA	0.36	0.25	1.46	0.15	-0.13	0.85
Basic	BC	TeloAA	-0.43	0.06	-6.75	<0.0001	-0.55	-0.30
Behavior	BC	TeloAA	-0.43	0.06	-6.78	<0.0001	-0.56	-0.31
Clinical	BC	TeloAA	-0.40	0.06	-6.47	<0.0001	-2.50	-1.33
Full	BC	TeloAA	-0.40	0.06	-6.46	<0.0001	-0.53	-0.28
